# Book Review

**DOI:** 10.1120/jacmp.v7i3.2289

**Published:** 2006-08-24

**Authors:** Edward F. Jackson

**Affiliations:** ^1^ UT MD Anderson Cancer Center 1515 Holcombe Blvd., Unit 056 Houston Texas 77030

## Abstract

*Imaging Systems for Medical Diagnostics: Fundamentals, Technical Solutions, Applications for Systems Applying Ionizing Radiation, Nuclear Magnetic Resonance and Ultrasound*, Arnulf Oppelt, Editor, Publicis Corporate Publishing, John Wiley and Sons Distributor, ISBN 3‐9578‐226‐2 (cloth) $145

Given the full title of this book, one would naturally assume that it must quite a tome. Coming in, by my count, at 21 chapters, 989 pages, 687 figures (many in color), and 821 references contributed by 85 authors and co‐authors, this text is certainly ambitious in its scope and in its attempt to provide current information on imaging modalities that undergo substantial changes more frequently than such texts can be written and published. The edition made available for review was published by Publicis Corporate Publishing in 2005 and was clearly supported by Siemens Medical Solutions. This fact is made clear throughout the text as all figures illustrating actual hardware do so with Siemens products and discussions of some specific acquisition techniques are “Siemens‐centric”. However, in several chapters the authors do make an effort to provide cross‐referencing of Siemens‐specific naming conventions to those conventions used by other major manufacturers of medial imaging equipment. In any case, such a focus to a specific vendor does not detract from the quality of the text.

While many JACMP readers, having never heard of Imaging Systems for Medical Diagnostics, will assume this is a new offering, it is actually the fourth edition of a text that was first distributed as a German language paperback in 1980. Two additional editions followed, with the last being produced in 1995. Of these previous editions, however, only the second edition was distributed in English (in 1990). Given the quality of this current edition, I would certainly expect to see a future edition.

The text is organized in five major parts, starting with the Principles of Image Processing and then moving to the Physics of Imaging, Image Reconstruction, Image Instrumentation, and finishing with Information Processing and Distribution. Within the Principles of Image Processing section, the authors discuss, in 5 chapters spanning 97 pages, the basics of sensory physiology and neural processing of visual information, subjective assessment of image quality (including ROC analysis), image rendering (segmentation, surface and volume rendering, etc.), image fusion (with applications to nuclear medicine, surgery, radiation oncology treatment planning, and interventional radiology), and navigation (with applications to image‐guided surgery and interventional radiology). Given the amount of material covered in a relatively short space, the authors do a very good job introducing the issues, giving relevant and timely examples, and providing adequate references for the reader to turn to for more detailed information. Transitioning from the basic principles of image processing to the physics behind medical imaging instrumentation, the authors provide an overview of the Physics of Imaging in 3 chapters spanning 94 pages covering topics in x‐ and γ‐ray physics (generation, interaction with matter, biological effects), magnetic resonance imaging (the basic NMR phenomenon, Bloch equations, relaxation mechanisms, nuclear induction, basic imaging and spectroscopy sequences), and ultrasound (field parameters, propagation, imaging principles, safety). As each of these topics can be the basis of an entire textbook, one would not expect the authors to be able to go into detail on each of these topics. Therefore, derivations of key results, for example, progress rapidly and would likely be difficult for the uninitiated to follow without supplementary reference material. (Not surprisingly, this is most apparent in the MRI chapter). Following this basic physics introduction, the authors provide a very brief two‐chapter, 28‐page, introduction to image reconstruction techniques, beginning with a discussion of contrast, spatial resolution, and noise (focused specifically on x‐ray imaging systems) and then introducing the more common reconstruction techniques (2D Fourier, filtered back‐projection, 3D projection reconstruction, etc.). Again, those who specialize in image reconstruction algorithms will find the treatment quite terse, and supplementary reference materials would certainly be necessary for a reader unfamiliar with image reconstruction techniques and image quality metrics.

The bulk of the text is allocated, reasonably, given the title, to Image Instrumentation. It is this section, 642 pages in eight chapters, that is, in this reviewer's opinion, the best in the book. It begins with a chapter on image displays (technologies, performance characterization), and then moves to chapters on x‐ray systems (tubes, generators, digital detectors, C‐arm cone‐beam systems, mammography), CT scanners (including multislice scanners and a brief introduction to cone beam CT), nuclear medicine systems (Anger cameras, SPECT, PET, and applications), MR systems (an overview of each subsystem, acquisition techniques including parallel imaging methods, motion correction/compensation, angiography, diffusion, fMRI, and spectroscopy, and applications), ultrasound systems (imaging modes, system architecture, transducer design, b‐mode imaging and applications including 3D, Doppler, harmonics and compounding, and contrast agents), special and hybrid imaging systems (portal imaging systems for radiation therapy, PET/CT,SPECT/CT, image‐guided therapy systems), and molecular imaging (basic introduction to imaging probes, amplification strategies, introductory modality‐specific applications). Throughout this section of the book, good use is made of high quality figures, many of them color, demonstrating modern clinical and research applications of the various modalities. Naturally, specialists in any particular modality discussed in this section will likely consider their particular modality's coverage too brief or too superficial. However, on balance, this section provides a good introduction to the instrumentation, with enough contemporary applications to allow the reader to appreciate the power of modern imaging systems.

The final section of the book, comprised of 3 chapters spanning 101 pages, is on Information Processing and Distribution and provides examples of software platforms (architectures, DICOM services, and medical enterprise application integration), computer‐aided detection and diagnosis, and hospital information systems (HIS architecture, electronic medical record, workflow, radiology information systems, PACS). It is in this section that the fact that this book is a product of Siemens Medical Solutions employees is perhaps most apparent as the “syngo” product line architecture is discussed rather extensively. However, choosing a single integration system is reasonable and this section of the book is, in fact, useful as a basic overview to HIS/RIS/PACS components and their integration.

In conclusion, the editor, authors, and co‐authors have, in this reviewer's opinion, taken on an immense challenge – to provide a text that covers topics ranging from image perception to the physics of medical imaging to image reconstruction techniques to instrumentation to image and information distribution. The back cover of the book states that the text is “intended as a handbook for students in biomedical engineering, for medical physicists, and for engineers working on medical technologies, as well as for lecturers at universities and engineering schools”. As such a handbook, the text achieves its goal. However, given the breadth of material covered, it is not surprising that certain sections are “light” on details. If used as a bioengineering or medical physics textbook at the graduate level, additional reference materials, some cited within the text, would be necessary. However, few books provide such a wealth of information, with high quality illustrations from contemporary applications, that is afforded by this text.

